# Web-Based Bereavement Care: A Systematic Review and Meta-Analysis

**DOI:** 10.3389/fpsyt.2020.00525

**Published:** 2020-06-24

**Authors:** Birgit Wagner, Nicole Rosenberg, Laura Hofmann, Ulrike Maass

**Affiliations:** ^1^Clinical Psychology and Psychotherapy, Medical School Berlin, Berlin, Germany; ^2^Clinical Psychology and Psychotherapy, University of Potsdam, Potsdam, Germany

**Keywords:** grief, bereavement, depression, post-traumatic stress disorder, Internet, e-health, intervention, psychotherapy

## Abstract

**Background:**

Web-based interventions have been introduced as novel and effective treatments for mental disorders and, in recent years, specifically for the bereaved. However, a systematic summary of the effectiveness of online interventions for people experiencing bereavement is still missing.

**Objective:**

A systematic literature search was conducted by four reviewers who reviewed and meta-analytically summarized the evidence for web-based interventions for bereaved people.

**Methods:**

Systematic searches (PubMed, Web of Science, PsycInfo, PsycArticles, Medline, and CINAHL) resulted in seven randomized controlled trials (*N* = 1,257) that addressed adults having experienced bereavement using internet-based interventions. We used random effects models to summarize treatment effects for between-group comparisons (treatment *vs.* control at post) and stability over time (post *vs.* follow-up).

**Results:**

All web-based interventions were based on cognitive behavioral therapy (CBT). In comparison with control groups, the interventions showed moderate (*g* = .54) to large effects (*g* = .86) for symptoms of grief and posttraumatic stress disorder (PTSD), respectively. The effect for depression was small (*g* = .44). All effects were stable over time. A higher number of treatment sessions achieved higher effects for grief symptoms and more individual feedback increased effects for depression. Other moderators (*i.e.* dropout rate, time since loss, exposure) did not significantly reduce moderate degrees of heterogeneity between the studies.

**Limitations:**

The number of includable studies was low in this review resulting to lower power for moderator analyses in particular.

**Conclusions:**

Overall, the results of web-based bereavement interventions are promising, and its low-threshold approach might reduce barriers to bereavement care. Nonetheless, future research should further examine potential moderators and specific treatment components (*e.g.* exposure, feedback) and compare interventions with active controls.

## Introduction

Grief after the loss of a significant person is a natural process, and most people adapt their grief gradually to their life after the death of a loved one. However, some people experience difficulties adjusting their grief over time. These difficulties can include separation distress, avoidance behavior, yearning for the deceased or a lack of acceptance of the loss (1). The fifth edition of the Diagnostic and Statistical Manual of Mental Disorders [DSM-5; (2)] included the diagnostic criteria of the Persistent Complex Bereavement Disorder (PCBD) as diagnosis requiring further research, and the 11th edition of the International Classification of Diseases [ICD-11; (3)] included the clinical diagnosis Prolonged Grief disorder (PGD). The main difference between these two criteria sets concerns the time criterion. The PCBD criteria require a functional impairment for at least 12 months after the loss of a significant person, while the PGD criteria require only 6 months of functional impairment. Boelen and Lenferink (4) analyzed in their study four additional existing criteria sets for pathological forms of grief and compared the six diagnostic criteria regarding symptom combination, prevalence, and dimensions. The results of their study indicate a variation in dimensions and symptom combinations and prevalence rates ranging from 10 to 20%. They conclude that the existing criteria sets do not identify the same diagnostic symptom cluster. Similar findings were found in a German treatment-seeking sample (5), showing larger prevalence rates for PGD (69%) compared to PCBD (48%). Studies have shown that pathological grief differs from other disorders such as depression and PTSD and described a distinct diagnosis (4). Nonetheless, there are important associations with depressive symptoms (*e.g.*, feelings of meaninglessness and worthlessness) as well as posttraumatic stress symptoms [*e.g.*, distressful remembrance of the traumatic death of a loved one; (6)].

Today the research on PGD implies not only that there is a strong need for sound and valid diagnostic criteria; there is also a great need for research for treatment strategies for the bereaved.

Previous meta-analyses and systematic reviews of the treatment effects of face-to-face grief interventions have shown inconsistent results (7–11). For example, whereas it was shown that preventive interventions (*i.e.* to prevent a worsening of normal grief processes) were largely ineffective [d = 0.03–0.16; (11, 12)], interventions that were specifically aimed at prolonged grief symptoms have yielded better treatment effects [d = 0.53; (11); see also (10, 12–14)]. The most recent meta-analysis analyzed 31 randomized controlled studies (8). The authors found small but significant effects for psychological interventions for grief symptoms at post-intervention (Hedge's *g* = 0.40). These effects could be maintained at follow-up. In addition to these reviews, treatment approaches that were based on cognitive–behavioral components yielded good treatment efficacy (15–18).

Irrespective of the clinical evidence, manualized interventions are still not routinely used in outpatient care, nor are they easily available to clients who suffer from PGD or belong to a high-risk group. Currently, therapists specializing in the treatment of PGD are still rare. The number of bereaved people seeking psychological treatment is relatively low, too. For example, Currow et al. (19) examined the nature of bereavement help-seeking following an expected death in a population-based survey (*N* = 6,034) in Australia, and only 1.5% reached out for help from a doctor or nurse; 2.2% contacted a grief counsellor, and 1.9% contacted a spiritual advisor. Another cross-sectional survey of young adults bereaved by suicide and other sudden deaths (*N* = 3,432) in the United Kingdom found that only 13% of the participants had received formal support from health professionals. The most common barriers to utilizing professional mental health support are the worry that it might be too painful to speak about the grief experience (20), the belief that it is too difficult to find help (21), or a fear of stigmatization, specifically for those bereaved by suicide (22).

In the past years, accumulating research has shown that internet-based interventions—particularly cognitive behavioral interventions—can be beneficial for most common mental health disorders, with treatment effects comparable to those of face-to-face treatments (23). Further, studies have shown that internet-based interventions for posttraumatic stress disorder (24, 25) yield medium to large treatment effects compared with a waitlist control group. The interventions are usually delivered in different forms, ranging from self-help treatments without a therapist's guidance to mainly text-based interventions with high therapist involvement (26–31). Web-based bereavement care has a number of advantages which can overcome typical obstacles to receiving support. For example, online interventions offer geographic independence and a widespread dissemination of treatments. In addition, they provide a user-friendly and flexible approach that is more responsive to the reality of most people living in today's digitalized societies. Finally, there is web-based support that can be relatively anonymous (*e.g.*, self-help apps or text-based programs) and can help bereaved patients overcome their initial shame or perceived feelings of stigma and might encourage them to confront themselves with feelings of guilt or disclosure of painful feelings.

In sum, there is growing evidence that psychological interventions have a positive effect on pathological grief symptoms, and there is also research showing the efficacy of internet-based interventions for mental disorders such as anxiety and affective disorders for a variety of populations. Considering that only a minority of bereaved people actively seek traditional forms of support [*e.g.*, (19, 32)], an additional digital dissemination of evidence-based bereavement care might reach even more mourners. With the growing interest of psychotherapy research for e-mental-health interventions, a review of web-based interventions for the bereaved is still missing.

The aim of this systematic review and meta-analysis is to investigate the effectiveness of web-based bereavement interventions compared with control groups in reducing symptoms of grief in adults. Further, we examine the effects of these interventions on PTSD or depression. Due to the fact that existing research has been based on different diagnostic criteria sets, this review does not limit its scope to ICD-11 defined PGD but also includes the other existing criteria sets of disturbed grief symptoms (4) that were, for example, defined as “complicated grief disorder” (33) or “prolonged grief disorder” (1).

## Methods

The Preferred Reporting Items for Systematic Reviews and Meta-Analyses statement (**Supplementary Table 1**; 34) was used as a guide for the literature search and reporting of results.

In PICOS terms (35), the current review addressed adults (≥18 years) who have experienced all types of losses of a significant person (Population). The main focus was the use of a web-based psychological treatment (Intervention) which was compared to a control group of any kind (Comparator). We focused on the results of measurements assessing grief symptoms as defined by the original study authors (Outcomes). Here grief did not necessarily have to be determined as the primary outcome in the original study, but the intervention should be aimed at grief. In addition, we examined PTSD and depression as outcome measurements. We included only randomized-controlled trials (Study design).

### Identification, Selection of Studies, and Data Extraction

We included all studies according to the following criteria: (a) publication in a peer-reviewed journal; (b) presence of a randomized controlled design; (c) use of an internet-based intervention; (d) participants lost someone through death (*i.e.* bereaved individuals); and (e) assessment of prolonged grief symptoms using (f) validated grief measurements. The following exclusion criteria were also applied: (a) presentation of secondary data only; and (b) investigation of a heterogeneous sample (*e.g.* individuals suffering from traumas other than loss) without presenting results for bereaved individuals separately.

The following databases were used for the literature search: PubMed, Web of Science, PsycInfo/PsycArticles, Medline, and CINAHL. The search included the terms [(“grief” OR “mourning” OR “bereaved” OR “bereavement” OR “death”) AND (“online” OR “internet” OR “computer” OR “web”) AND (“intervention” OR “therapy” OR “self-help” OR “treatment” OR “program” OR “expressive writing”) AND (“randomized” OR “randomized” OR “controlled”)]. We searched the titles and abstracts of relevant articles that were written in English and were published between 1990 and January 17, 2020. In addition, we screened the reference lists of previous reviews and meta-analyses dealing with the issue of internet-based interventions and bereavement/trauma for relevant articles.

Four authors (BW, UM, LH, NR) extracted the following variables: author(s), publication year, title, country, time points of assessment, conditions, outcomes measures, sample size, characteristics of participants and interventions, type of analysis, means and standard deviations of outcome measures (i.e. PGD, depression, PTSD), and characteristics of dropouts.

The quality of the included studies was evaluated according to the list of Van Tulder et al. (36), which was based on recommendations by the Cochrane Collaboration Back Review Group (rating scale: yes, no, or unclear) and which the authors applied for their review on internet-based interventions. We eliminated one item because it was not suitable for this review and would have biased the results. The methodological quality of a study was rated good if it achieved a total yes-score ≥ 8 (*i.e.* met at least two thirds of the criteria). The rating was implemented independently by two of the authors (NR, BW) and was discussed in the case of dissent.

### Effect Size Calculation

The meta-analytical summary of the effect sizes was done using RStudio 1.1.456 (37) and the metafor package (38) for two comparisons: (1) a between-group (treatment *versus* control group) at the post assessment, and (2) a within-group design to estimate the stability of the effects of the treatment from post to follow-up assessments. When studies reported several follow-up assessments, we took the one that was closer to the mean time interval. The mean differences between the means of the symptom levels were determined using the results of the studies' intention to treat (ITT) or per protocol (PP) data if ITT were not available. Hedge's *g* was used as the standardized effect size (ES) to correct for sample size differences (39) and was classified as small (*g* < 0.50), moderate (0.50 ≤ *g* ≤ 0.80), or large (*g* > 0.80) (40). Multiple assessments of the same outcome were combined to control for dependencies within the data. Heterogeneity (*i.e.* variation in ESs between the studies) using the Q-statistic and the I2 index, which indicates low (25%), moderate (50%), or high (75%) levels (41). In cases of at least moderate heterogeneity, we conducted separate meta-regressions to identify potential sources of this heterogeneity (42). The following moderators were considered relevant to explain potential differences in intervention characteristics: (a) dropout rate in the treatment and control group, (b) time since loss in months and number of treatment sessions, (c) the extent of personal therapeutic feedback during treatment (no feedback = 0, informative feedback = 1, personal therapeutic feedback = 2), and (d) the use of exposure, cognitive reappraisal, or behavioral activation within the treatment (each: not applied = 0, applied = 1). All analyses used random-effects models that allowed the amount of residual heterogeneity to be different in each study. A power analysis for random effects models (42) revealed that 6 studies are needed to achieve a power of.84 expecting a moderate mean ES of Hedge's *g* = 0.50 for grief in studies with at least 20 participants per group and moderate heterogeneity (11, 24).

## Results

**Figure 1:**
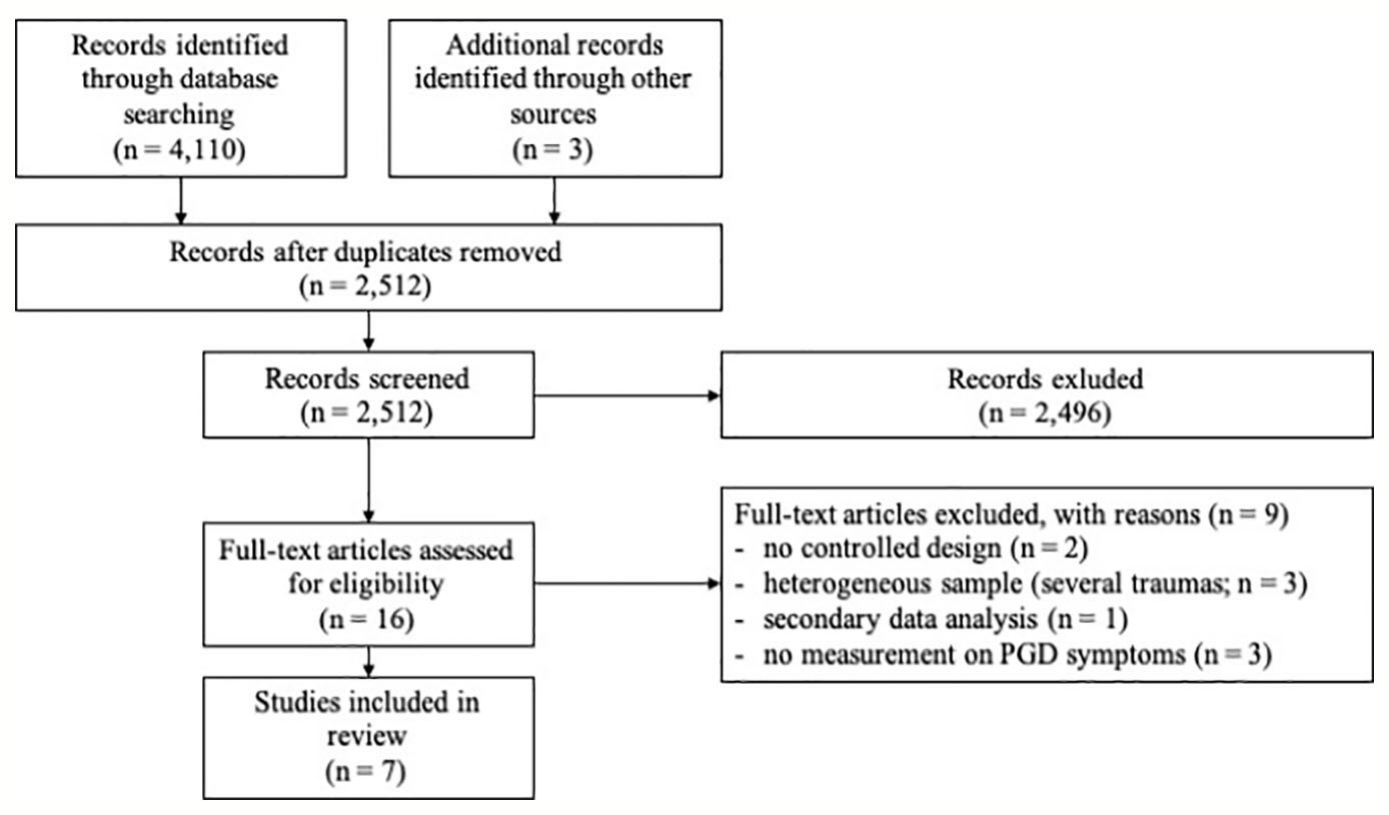
Study flow diagram, showing the results of the literature search for this current review.

**Figure 1** shows the selection process. Seven studies were included in this review.

### Study Characteristics and Methodological Quality

The study characteristics are presented in **Table 1**. One study (43) provided two between-group comparisons because the authors compared two conditions with each other (exposure *vs.* behavioral activation) and against a control group. The studies of Wagner (18) and Wagner and Maercker (44) were based on the same trial but focused on different time points: posttreatment symptoms versus follow-up. Consequently, there were seven studies based on six trials yielding seven comparisons at post and follow-up for grief.

**Table 1:** Study characteristics.

Study	Eisma et al. (43)	Kersting et al. (46)	Kersting et al. (45)	Litz et al. (48)	van der Houwen et al. (47)	Wagner et al. (18)	Wagner and Maercker (44)^a^
**Condition**	1. Exposure (EX)	1. Treatment	1. Treatment	1. Treatment	1. Treatment	1. Treatment	1. Treatment
	2. Behavioral activation (BA)	2. WL control	2. WL control	2. WL control	2. WL control	2. WL control	2. WL control
	3. WL control						
**Outcome**	Prolonged grief (ICG-R > 25), posttraumatic stress, anxiety, grief, depressive rumination	Prolonged grief symptoms, posttraumatic stress, depression, somatization, anxiety, general mental health	Prolonged grief (score > 36 on separation and traumatic distress), Posttraumatic stress, depression, anxiety, general mental health	Prolonged grief (PG-13 > 23), depression, posttraumatic stress, anxiety	Prolonged grief symptoms, depression, positive mood, emotional loneliness	Prolonged grief (predetermined cut-off scores): failure to adapt, Intrusion, avoidance, depression, anxiety, general mental, physical health	Prolonged grief (predetermined cut-off scores): failure to adapt, Intrusion, avoidance, depression, anxiety, general mental, physical health
**Measurement**
Grief	ICG-R	ICG	ICG	PG-13	9 items based on the criteria for complicated grief ^b^	5 items from the revised symptom list for complicated grief ^c^	5 items from the revised symptom list for complicated grief ^d^
PTSD	PSS	IES	IES-R	PCL-b	–	IES-I / IES-A	IES-I / IES-A
Depression	HADS	BSI	BSI	BSI	CED-D	BSI	BSI
**Sessions / duration^e^**	6 / 6-8	10 / 5	10 / 5	18 / 6	5 / 5	10 / 5	10 / 5
**Assessment**	Pre, Post, FU (3)^e^	Pre, Post, FU (3)^e^	Pre, Post, FU(3, 12)^e^	Pre, Post, FU (1.5, 3)^e^	Pre, Post, FU (3)^e^	Pre, Post, FU (3)^e^	FU (18)^e^
**Sample size**							
- Treatment (Rand/Post/FU)	EX: 18 / 15 / 12 BA: 17 / 11 / 11	48 / 33 / 29	115 / 99 / 85, 45	43 / 32 / 31, 31	460 / 201 / 190	29 / 26 / 25	29 / 26 / 22
- WL-control (Rand/Post/FU)	12 / 10 / 10	35 / 26	113 / 100	44 / 42 / 35, 35	297 / 254 / 217	26 / 25	26 / 25
**Dropout rates (treatment : control)**	33.3% (EX), 58.8% (BA) : 16.7%	26.7% : 21.2%	13.9% : 11.5%	22.0% : 2.3%	52.0% : 14.5%	10.3% : 3.8%	15.4%^f^

GP, general population; CBT, cognitive behavioral therapy; WL control, waitlist control; Rand, randomized; Post, post treatment; FU, completed follow-up assessment; EX, exposure; BA, behavioral activation; Pre, pre treatment; ICG, Inventory of Complicated Grief [ICG-R; (49); ICG; (50)]; PG-13, Prolonged Grief Scale (1); PSS, PTSD Symptom Scale (51); IES, Impact of Event Scale; IES-I,Intrusion; IES-A, Avoidance [IES; (52); IES-R; (53)]; PCL-C, PTSD Checklist - civilian version (54); HADS, Hospital Anxiety and Depression Scale (55); BSI, Brief Symptom Inventory (56); BDI-II, Beck Depression Inventory-II (57); CES-D, Center for Epidemiological Studies-Depression Scale (58).

^a^1.5-year follow-up data based on the study by Wagner et al. (18). ^b^proposed for DSM-V. ^c^(59): trouble sleeping, feeling worthless, an altered sense of future, and feeling lonely or empty. ^d^number of sessions / duration in weeks. ^e^in months after the post assessment. ^f^based on participants who dropped out from the pre treatment in the study by Wagner et al. (18) to the 18-month follow-up in Wagner and Maercker (44).

All studies assessed PGD, depression, and PTSD symptoms in response to the loss (see **Table 1** for the according measurements).

The methodological quality of the included studies varied, with no study meeting all 12 criteria (see **Table 2**). Four studies (50%; 43, 45–47) were rated as having good methodological quality.

**Table 2:** Methodological quality of included studies.

Study	Eisma et al. (43)	Kersting et al. (46)	Kersting et al. (45)	Litz et al. (48)	van der Houwen et al. (47)	Wagner et al. (18)	Wagner and Maercker (44)^a^
Were the eligibility criteria specified?	Yes	Yes	Yes	Yes	Yes	Yes	Yes
Was the method of randomization described?	Yes	Yes	Yes	No	No	No	No
Was the random allocation concealed?^b^	Unclear	Unclear	Unclear	Unclear	Unclear	Unclear	Unclear
Were the groups similar at baseline regarding important prognostic indicators?	No	Yes	No	No	Yes	Yes	Yes
Were both the index and the control interventions explicitly described?	Yes	Yes	Yes	Yes	Yes	Yes	Yes
Was the outcome assessor blinded to the interventions?	Unclear	Unclear	No	Unclear	Unclear	Unclear	Unclear
Was the dropout rate described, and were the characteristics of the dropouts compared with the completers of the study?	Yes	Yes	Yes	Yes	Yes	No	Yes
Was a long-term follow-up measurement performed?^c^	No	No	Yes	No	Yes	No	Yes
Was the timing of the outcome measurements in both groups comparable?	Yes	Yes	Yes	No	Yes	Yes	No^d^
Was the sample size for each group described by means of a power calculation?	Yes	Yes	No	No	No	No	No
Did the analysis include an intention-to-treat analysis?	Yes	Yes	Yes	Yes	Yes	Yes	No
Were the point estimates and measures of variability presented for the primary outcome measures?	Yes^e^	Yes	Yes	Yes	Yes	Yes	Yes
**Total number of criteria fulfilled**	**8**	**9**	**8**	**5**	**8**	**6**	**6**

CG, control group; FU, follow-up.

^a^1.5-year follow-up data based on the study by Wagner et al. (18). ^b^“Was the assignment generated by an independent person not responsible for determining the eligibility of the patients?” ^c^outcomes measured ≥ 6 months after randomization. ^d^No control group at follow-up. ^e^Available as a supplement.

Six studies (18, 43, 45–48) reported results from ITT analyses. One study (48) applied linear mixed models using REML, two studies applied multilevel analyses (43, 47), and three studies used the last observation carried forward approach to handle missing data (18, 45, 46). To calculate the ES for our review, we referred to the corresponding sample sizes per protocol (PP) except for Kersting et al. (45), who reported means from ITT data, and Wagner et al. (18), who reported means for completers only.

### Samples

The studies included a total of *N* = 1,257 randomized participants from the general population. **Table 3** presents the sample's characteristics. Women comprised between 68 and 100% of the samples. The average age of the total sample was 41.7 years. Overall, participants tended to be well-educated (i.e., college and university degrees), with five studies indicating that the education level of most of their participants was high.

**Table 3:** Sample characteristics.

Study	Eisma et al. (43)	Kersting et al. (46)	Kersting et al. (45)	Litz et al. (48)	van der Houwen et al. (47)	Wagner et al. (18)	Wagner and Maercker (44)^a^
Country	Netherlands	Germany	Germany	USA	USA, UK^b^	Germany^c^	Germany^c^
Population	GP	GP	GP	GP	GP	GP	GP
Subjects	Subjects with elevated levels of complicated grief^d^ and elevated grief rumination^e^	Mothers after pregnancy loss	Parents after pregnancy loss	Bereaved caregivers with elevated levels of prolonged grief^f^	Bereaved subjects significantly distressed by the loss	Bereaved individuals with symptoms of intrusion, avoidance, and maladaptive behavior^g^	Bereaved individuals with symptoms of intrusion, avoidance, and maladaptive behavior^g^
**Age^h^*M (SD)***	45.7 (12.9)	34.3 (5.3)	34.2 (5.15)	55.4 (10.33)	43.2 (11.0)	37.6 (10.3)	36.0 (11)
**Female in %**	91.5	100	92.1	67.9	93.5	92.3	88
**Education in %^i^**							
- Low	40.4	9.0	4.4	1.2	16.8	15.6	
- Medium		44.9	12.7	31.1	47.0	39.2	41
- High	59.6	46.2	82.9	67.8	36.2	31.2	31
**Relationship to the deceased in %**							
- Spouse/Partner	40.4^j^	–	–	82.1	30.4	10	10
- Child	–	100	100	4.7	42.5	62	61
- Parent	–	–	–	7.3	16.6	6	10
- Parent-in-law	–	–	–	–	–	–	–
- Grandparent	–	–	–	–	–	–	–
- Sibling	–	–	–	2.5	10.4	4	3
- Aunt/uncle	–	–	–	–	–	–	–
- Relative	–	–	–	2.5	–	4	16^k^
- Friend	–	–	–	1.2	–	14	–
**Type of loss in %**							
- Expected natural death	Cause of death was categorized as nonviolent (78.7%) or violent (22.8%)	–	–	100	–	–	–
- Natural death		–	–	–	65.8	36	42
- Pregnancy loss		100	100	–	–	–	–
- Stillbirth/SID		–	–	–	–	18	16
- Accident		–	–	–	22.1^l^	26	23
- Suicide		–	–	–	12.2	20^m^	19^m^
**Time since loss in months M (SD), [range]**	31.0 (45.1)	15.4 (27.4), [1-144]	9.93 (24.11)	8.38 (2.97)	40.44 (62.88)	55.2 (78.6), [14-348]	48 (60), [14-192]

SID, sudden infant death; GP, general population.

^a^1.5-year follow-up data based on the study by Wagner et al. (6).

^b^Including English speakers living in other countries. ^c^Including German speakers living in other countries. ^d^Score on the Inventory of Complicated Grief > 25. ^e^Score on the Utrecht Grief Rumination Scale > 40. ^f^Score on the Prolonged Grief Scale > 22 as well as functional impairment in social, occupational, or household responsibilities. ^g^Caused by the death of a significant other within predetermined cut-off scores that were not described in the study. ^h^In years. ^i^Includes incomplete information. The labels “low”, “medium”, and “high” refer to the classification used by the original study authors, which are not always explained in more detail. Generally, low education relates to the early school forms (i.e., primary school, high school or vocational school) and higher education to the final stages in college or university (e.g., masters or doctoral degree). ^j^Relationship of the remaining 59.6% could be children, siblings, or parents. ^k^Including relatives and friends. ^l^Including accident and homicide. ^m^Including suicide and homicide.

Two of the included studies addressed only mothers (46) and parents (45), respectively, who had lost a child during pregnancy, whereas one study (48) included only people who had experienced the expected natural death of someone. The causes of death in the remaining studies were (unexpected) natural death, stillbirth, sudden infant death, or death by accident, suicide, or homicide. Time since loss ranged from 8.38 to 55.2 months, with a mean of 26.7 months, indicating great variability for this variable.

All studies used measurements that explicitly addressed symptoms that were labeled as “complicated” or “prolonged” grief or grief disorder [see (4) for a comparison of different criteria sets for PGD]. As **Table 1** displays, only two studies did not apply a cut-off score to indicate higher levels of PGD (46, 47).

### Characteristics of the Interventions

**Table 4** provides an overview of the characteristics of the different intervention programs. Three studies included minimal therapeutic support, such as explanations regarding homework and minor logistical help (43, 47, 48). Four studies involved individualized feedback after most treatment modules (18, 44–46). Further, five studies used structured writing assignments (18, 44–47) based on the Pennebaker paradigm (62). All of the included studies used an approach based on cognitive behavioral therapy (CBT).

**Table 4:** Characteristics of the intervention programs

Study	Eisma et al. (43)	Kersting et al. (46)	Kersting et al. (45)	Litz et al. (48)	van der Houwen et al. (47)	Wagner et al. (18)	Wagner and Maercker (44)^a^
**Therapeutic approach**	CBT	CBT	CBT	CBT	CBT	CBT	CBT
**Content / Modules**	e-mailed homework assignments; Exposure^b^: writing assignments, imaginal and/or in vivo exposure exercises; Activation^c^: 7-day activity diary, engagement in value-based activities	Writing assignments^d^: (1) 4 self-confrontation (describe the traumatic loss and its circumstances), (2) 4 x cognitive restructuring (supportive letter to a hypothetical friend, develop new perspectives on the loss), (3) 2x social sharing (symbolic farewell letter)	Writing assignments^d^: (1) 4 self-confrontation (describe the traumatic loss and its circumstances), (2) 4 x cognitive restructuring (supportive letter to a hypothetical friend, develop new perspectives on the loss), (3) 2x social sharing (symbolic farewell letter)	HEAL intervention: (1) psycho-education about loss and grief, (2) instruction in stress management and other coping skills, (3) behavioral activation (self-care + social re-engagement), (4) accommodation of loss + goal achievement, (5) relapse prevention	Writing assignments^d^: (1) 2x exposure (most-distressing aspects of the loss), (2) 2x cognitive reappraisal (identify unhelpful thoughts, develop helpful thoughts; letter to a hypothetical friend), (3) 1x integration, and restoration (letter to deceased from future)	Writing assignments^d^: (1) 4x exposure (circumstances of death, most-distressing aspects of the loss), (2) cognitive reappraisal (letter to a hypothetical friend, develop new role, rituals, positive resources), (3) integration, and restoration (letter to significant person, most important memories, coping with death, changes)	Writing assignments^d^: (1) 4x exposure (circumstances of death, most-distressing aspects of the loss), (2) cognitive reappraisal (letter to a hypothetical friend, develop new role, rituals, positive resources), (3) integration, and restoration (letter to significant person, most important memories, coping with death, changes)
**Exposure**	Yes	Yes	Yes	No	Yes	Yes	Yes
**Cognitive restructuring**	No	Yes	Yes	No	Yes	Yes	Yes
**Behavioral activation**	Yes	No	No	Yes	No	No	No
**Sessions / Duration**	6 sessions / 6-8 weeks	10 sessions à 45 min / 5 weeks	10 sessions à 45 min / 5 weeks	18 sessions à 20 min / 6 weeks	5 sessions / 5 weeks	10 sessions à 45 min / 5 weeks	10 sessions à 45 min / 5 weeks
**Frequency of contact**	After each assignment; intake interview by telephone	Once per week; telephone call before treatment^e^	Once per week; telephone call before treatment^e^	Periodically via email, on average 0.6 emails and 0.24 calls per week; single phone call at the beginning of treatment	Once per week for instruction	Twice per week	Twice per week
**Therapeutic feedback**	Informative (explaining homework and maximizing treatment adherence)	Individual written feedback, instructions for the next writing assignment	Individual written feedback, instructions for the next writing assignment	Informative (information about program)	Informative (general guidelines for writing), no feedback on writing assignments	Individual written feedback every 2^nd^ essay, instructions for the next writing assignment	Individual written feedback, every 2^nd^ essay, instructions for the next writing assignment
**Manual**	Yes	Yes	Yes	Yes	Yes	Yes	Yes
**Multimedia**	No	No	No	No	No	No	No
**Reminder**	No	No	No	Yes	Yes	No	No

CBT, cognitive behavioral therapy.

^a^1.5-year follow-up data based on the study by Wagner et al. (6). ^b^Based on Boelen et al. (15). ^c^Based on Lejuez et al. (60). ^d^Based on Lange et al. (61). ^e^For applicants who scored high on any of the screening instruments to check for worsening of symptoms.

Regarding the content of the programs, six studies used a manual and included an exposure module (18, 43–47), five studies involved cognitive reappraisal (including integration and restoration, also called social sharing) (18, 44–47), and two studies used behavioral activation modules (43, 48). Five studies were based on an adjusted protocol for the treatment of posttraumatic stress disorder through the internet by Lange et al. (63).

### Dropouts

We defined dropouts as all participants who completed pre-assessments but did not complete the treatment (or waitlist period). The rates of dropouts (**Table 1**) ranged from 10.3 (18) to 58.8% (43).

All included studies provided some information about the characteristics of dropouts, except for Wagner et al. (18). Eisma et al. (43) reported no significant differences between completers and dropouts on any of the assessed variables (demographic, loss-related, symptom, and rumination). In the study by Kersting et al. (46), time since loss was significantly longer for dropouts. By contrast, time since loss was shorter for Kersting et al.'s (45) dropouts, who were also younger and lost their child significantly earlier in pregnancy (the study investigated pregnancy loss). Litz et al. (48) reported that their dropouts received more average weekly emails from the therapist during the program and that they were more often employed full-time than completers. Dropouts differed on several variables in the study by van der Houwen et al. (47): They were younger, had lower levels of education, experienced more grief and were more likely to be in the treatment condition.

### Effect Sizes

**Figure 2:**
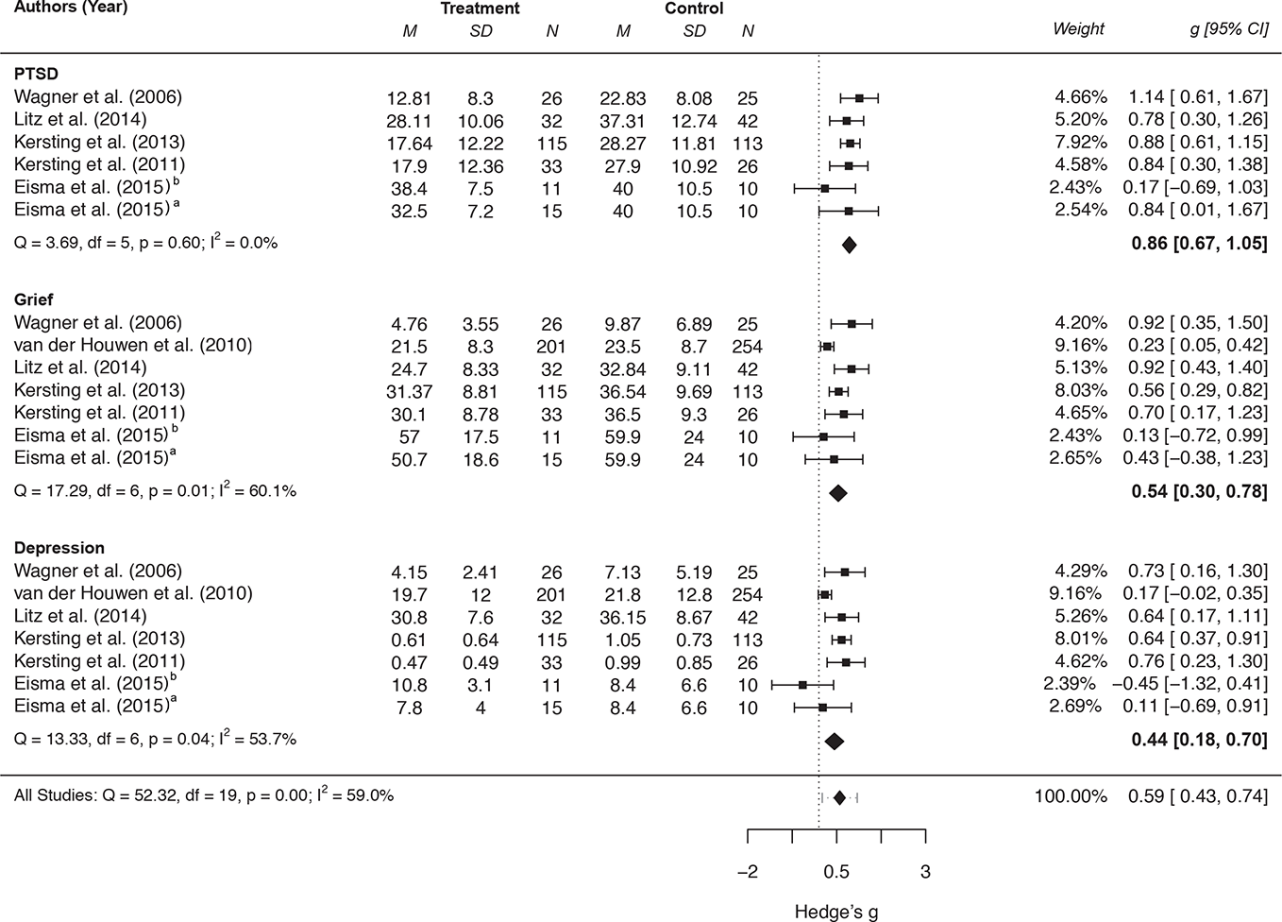
Forest plot of between-group effect sizes of internet-based interventions for PGD, depression and PTSD symptoms. ^a^Comparison between Exposure-based treatment and waitlist control group. ^b^Comparison between Behavioral activation and waitlist control group.

The results for the between-group comparisons are displayed in **Figure 2**. The overall effect on grief symptoms was moderate and significant with moderate to high heterogeneity between the studies. The ESs for PTSD^1^ and depression were almost moderate (depression) to large (PTSD) and statistically significant with low (PTSD) and high (depression) heterogeneity.

All effects remained stable from post-assessment to follow-up (3 months) with low (depression) to moderate (grief, PTSD) heterogeneity between the studies: (a) grief: *k* = 7, Hedge's *g* = 0.17, 95% CI [−0.03, 0.36], *p* = .094, *I*^2^ = 34.1%, *Q*(6) = 7.69, *p* = .261, (b) PTSD: *k* = 6, Hedge's *g* = 0.17, 95% CI [−0.06, 0.40], *p* = .154, *I*^2^ = 27.1%, *Q*(5) = 5.85, *p* = .321, and (c) depression: *k* = 7, Hedge's *g* = 0.07, 95% CI [−0.07, 0.20], *p* = .324, *I*^2^ <.01%, *Q*(6) = 1.54, *p* = .957.

There were only two moderators with a significant impact on the ESs (*p*s < 0.05, **Table 5**) and significantly reduced heterogeneity at post-assessment: A higher number of therapeutic sessions were associated with a higher ES for grief, and more individual feedback increased the ES for depression.

**Table 5:** Meta-regressions to test for the influence of moderators in models with moderate to high heterogeneities.

	Between-Group Comparison						Stability (Post-Follow Up)					
	Grief			Depression			Grief			PTSD		
	*B*	*95%CI*	*I^2^*	*B*	*95%CI*	*I^2^*	*B*	*95%CI*	*I^2^*	*B*	*95%CI*	*I^2^*
Dropout TG^a^	−0.005	[−0.02, 0.01]	8.38	−0.02	[−0.03, −0.002]	<0.01	<−0.001	[−0.02, 0.02]	35.5	0.01	[−0.02, 0.03]	49.1
Dropout CG^a^	−0.01	[−0.04, 0.02]		−0.01	[−0.02, 0.04]		−0.004	[−0.04, 0.03]		−0.004	[−0.05, 0.04]	
Time s. loss^b^	0.004	[−0.01, 0.02]	<.01	<−0.001	[−0.02, 0.02]	38.8	−0.01	[−0.03, 0.001]	<0.01	−0.01	[−0.02, 0.01]	14.1
No. sessions	0.07**	**[0.02, 0.12]**		0.05	[−0.02, 0.12]		−0.02	[−0.07, 0.03]		−0.04	[−0.11, 0.03]	
Feedback^c^	0.11	[−0.15, 0.37]	44.7	0.20*	**[0.002, 0.39]**	21.5	0.04	[−0.17, 0.26]	30.5	0.03	[−0.30, 0.36]	33.9
Exposure^d^	−0.16	[−0.76, 0.44]	55.1	0.21	[v0.48, 0.91]	69.4	−0.09	[−0.63, 0.46]	41.7	−0.04	[−0.63, 0.55]	36.6
Reappraisal	−0.08	[−0.64, 0.48]	57.6	0.30	[−0.32, 0.92]	68.8	−0.08	[−0.56, 0.41]	41.8	−0.003	[−0.40, 0.40]	<0.01
Activation	0.08	[−0.48, 0.64]	57.6	−0.30	[−0.92, 0.32]	68.8	0.08	[−0.41, 0.56]	41.8	0.003	[0.40, −0.40]	<0.01

TG, treatment group; CG, control group. Significant effects are in bold (p < 0.01). ^a^In percent, ^b^in months, ^c^variable was treated as continuous on a rating scale of 0 = no feedback, 1 = informative feedback, and 2 = personal therapeutic feedback, ^d^0 = no exposure, 1 = feedback. I^2^ = residual heterogeneity.

*p < 0.05. **p < 0.01.

Potential outliers for all outcomes were detected using influential case diagnostics (see **Supplementary Table 2**) Viechtbauer and Cheung (64), which marked two studies: van der Houwen et al., (47) at post-assessment (grief and depression) and Kersting et al. (45) at follow-up (grief and PTSD). While the results did not substantially change after recalculating the meta-analyses without Kersting et al.'s study, the ESs increased to a moderate degree and the heterogeneities decreased to zero after recalculating the analyses without van der Houwen et al.'s study (see **Supplementary Table 3**) (65). Publication bias was not inspected because the analysis included fewer than 10 studies and heterogeneity was often greater than 10 (66).

## Discussion

The present review systematically evaluated the efficacy of web-based bereavement care interventions for bereaved people with higher levels of disturbed grief, based on seven RCTs using active or waitlist control groups. The effects of the web-based interventions on grief reduction were promising with moderate ESs (Hedge's *g_between_* = 0.54), which was also stable from post to 3-months follow-up assessment. Overall, the results are in line with an earlier review of face-to-face interventions for grief (11) which yielded an effect size of 0.53. However, the most recent review (8) found only small effects for bereavement interventions, particularly when considering publication bias (*g* = 0.31). One explanation for the diverging results might be that the present review explicitly focused on online interventions while the Johannsen et al. (8) combined face-to-face and online treatments. Another reason might lie in the slightly different definitions of grief and the assessment of its pathological levels. For example, Johannsen et al. (8) included only studies that assessed grief using one of the versions of the ICG-R or PG-13. Facing similar decisions regarding definition and assessment, this review relied on the definitions of prolonged grief that was presented by the original authors of the included studies. With the new ICD-11 criteria and the use of consistent measurements, it should be possible to reduce variance between studies resulting from variance in outcome assessments in the future. Finally, the differences might also be due to different methodological approaches. For example, Johannsen calculated their effect sizes considering pre-post differences while this review based its estimation on postscores. Nonetheless, it is noteworthy that despite these different methodological approaches, the main conclusions from the existing reviews still seem to be comparable: Psychological treatment for pathological grief in general, and based on this review delivered *via* online-based formats in particular, is effective, but it is not yet as effective as it is in treating online, for example, panic or social anxiety disorders, given the absolute numbers of ESs (67).

The largest and most robust effect, however, was found for grief-related PTSD symptoms (Hedge's *g_between_* = 0.86) which also remained stable over time. These results are generally in line with research demonstrating the effectiveness of e-mental health approaches in the reduction of PTSD (24, 68). The result that interventions had stronger effects on posttraumatic symptoms than on prolonged grief had already been noted previously (8) but still needs clarification. One reason could be that the disturbed grief and PTSD have “less clear boundaries” (6, p. 2446). In fact, the majority of the current treatment protocols (*i.e.*, 86% of the included studies) are largely tailored to treat distressing memories that are associated with the loss and reduce avoidance using exposure-elements. Distressing remembrance is a cluster that shows the largest overlap between PCBD and PTSD (6). Thus, the current treatment protocols might address mutual grief and posttraumatic stress symptoms more than they address the specific symptoms of prolonged grief [*i.e.*, role confusion, meaninglessness, and loneliness; (6)].

The weakest effect was found for depression (Hedge's *g_between_* = 0.44), which is still comparable to the effects of general internet-based treatments for depression [(69–71); but see *e.g.*, (72) for larger effects] and the effects of general PGD interventions for secondary depression outcomes (8). There might be two reasons for the smaller effects on depression. First, there was high heterogeneity in our results that might partly be explained by the variability in the extent of therapeutic feedback. As our moderator analyses showed, studies with more personal feedback were more likely to report higher ESs for depression. Previous studies also reported that individualized internet interventions achieve higher outcomes compared to pure self-help or no guided tools (73). However, because our meta-regressions were somewhat underpowered, we interpret the results for depression cautiously with the outlook that more studies are needed to disentangle the associations between interventions, depression, and feedback. Second, given that major depression and PCBD are more distinct categories than PTSD and PBCD (6), the comparably smaller ESs might reflect the narrow focus of current treatment protocols to grief and not depression. However, because there is still overlap between both diagnoses with respect to, for example, worthlessness and guilt, the applied treatment concepts might still address some parts of depressive symptoms. Again, Johannsen et al. (8) also found no significant effect of bereavement treatments on depression and hypothesized that there might be no “spill-over effect of grief interventions on other psychological morbidities” (p. 78). This raises the question what kind of treatment ingredients are currently missing in treatment protocols that would address depressive symptoms more directly. Eisma et al. (43) examined a pure behavioral activation module but did not yield sufficient ES for depression. However, it would be interesting to combine writing assignments using exposure and cognitive reappraisal [*e.g.*, based on the approach by (50)] with behavioral activation.

Considering the attrition rates of the reviewed treatments, our results showed that, generally, the average attrition rate of 27% is comparable with other internet-based treatment studies (74). However, one interesting finding was the wide range of dropout rates between the treatment groups (*i.e.*, 10 to 58%). One factor that might contribute to this range could be the degree of guidance and personal feedback, because the highest dropout rates were found in treatments that offered only informative feedback (43, 47). These studies also mentioned that some participants dropped out because the intervention was “too impersonal”, or that the “treatment did not help”, “was too difficult”, or that they had “no confidence” in it [(43), p. 5; 47, p. 362]. The behavioral activation group in Eisma et al.'s study, for example, had the highest dropout rate, which might further point to difficulties that are associated with implementing behavioral changes by oneself. In addition, the average dropout rate for studies providing informative feedback was 41% (SD = 17) and only 17% (SD = 15) for studies providing personal feedback. Previous research suggested the importance of guidance in internet-based interventions for their efficacy and its superiority on adherence over completely unguided interventions (75, 76). This does not necessarily mean that the treatment concepts are ineffective but that additional factors (*i.e.*, guidance) are needed to facilitate change. The results of the outlier analysis similarly point to this direction, because, after excluding van der Houwen et al.'s study, heterogeneity between the studies was reduced and the ESs for grief and depression rose to a moderate level. This study applied writing assignments including exposure and reappraisal elements. Both seemed to be effective in the other included studies, but the intervention was very short (*i.e.*, five sessions) and dispensed with personal feedback. Thus, the dropout rate was high.

Similar to the range of dropouts, there was a large range between the number of sessions across studies (*i.e.*, five to 18). The moderator analyses showed that a higher number of treatment sessions were associated with higher effect sizes for grief. It is noticeable that the interventions with the highest dropout rates (43, 47) also had the lowest number of sessions (*i.e.*, six and five) and the lowest personal support. Again, this finding might also add to the hypothesis that participants suffering from PGD need more personal support over a longer period of time.

In sum, the interpretation of the present results on web-based bereavement care are mostly in line with previous research on face-to-face treatments: While online treatment is largely effective in treating PTSD symptoms, its effect on grief symptoms themselves is comparably lower, although moderate and stable over time. The effects on depression, however, are less clear and tend to be small.

### Limitations

A number of limitations of this review need to be addressed. First, only a small number of studies could be included in this review. E-mental health for the bereaved is still in its infancy, and further randomized controlled trials are needed. Although we reached the required number of at least seven studies to achieve adequate power, the number of included studies varied depending on the specific comparison and outcome (*i.e.* for meta-regressions). Also, not all studies included 20 participants per study group (43). It remains unclear whether the nonsignificant results for study characteristics (*e.g.* influence of exposure *vs.* no exposure) are true effects or whether our analyses were underpowered (42, 77).

Second, because all interventions were based on CBTs and almost all used exposure modules, we cannot make specific conclusions about their effectiveness compared to other treatment approaches. Nonetheless, they seem promising, since memories of the death and its circumstances are often experienced as very distressful and are therefore avoided. In line with this assumption is the rationale and effectiveness of face-to-face exposure-based treatments for PTSD (78) and PGD symptoms (15, 79). Interestingly, Eisma et al.'s (43) behavioral activity intervention did not reveal significant effects on grief-related PTSD, but the exposure group did. However, because of the very small sample sizes in each group (11 vs. 10 participants at the post assessment) and because of the significant baseline differences, the results were not considered in the effect size calculations for this review.

Third, the fact that in most studies, different types of losses were addressed (*e.g.* parental loss, bereaved parents, suicide bereavement) complicates the process of reaching a reliable conclusion.

Finally, this group of bereaved people included in this meta-analysis might not reflect the naturalistic setting in the treatment of clients with disturbed grief symptoms who are traditionally looking for help in self-help groups and who might additionally suffer from comorbid disorders, increased suicidality, and alcohol and substance abuse. Consequently, effectiveness studies should be conducted to provide better knowledge about the generalization of previous results from randomized controlled trials.

### Future Directions and Practical Implications

Since this review included all studies that measured PGD symptoms on a continuous scale, future studies might address PGD diagnosed according to one validated sound diagnostic criterion. Further, the identification of relevant moderators, such as symptom severity, type of loss, length of treatment, and amount of therapist support is needed to improve treatment outcomes from web-based interventions. Further, randomized controlled trials should involve active control groups, such as face-to-face groups or self-help interventions and compare writing assignments directly to other online-approaches.

Generally, there is a great need to understand the help-seeking patterns of bereaved people depending on gender, type of loss (*e.g.* suicide, illness), age group (*e.g.* adolescents, the elderly), and relationship to the deceased (*e.g.* child, parent, sibling). Further, dismantling studies should be conducted as well as blended care interventions, which could include internet-based interventions in existing psychosocial support (*e.g.* self-help groups). A challenging task might be the inclusion and treatment of less-educated participants in internet-based interventions because, so far, they are reaching a better-educated group of people (80).

In addition, future research studies should examine the underlying mechanisms of effective treatments. For example, it is worth mentioning that all interventions [except the behavioral activation group in (43)] included extensive structured writing assignments, which might be one promising treatment approach that is applicable to both, online and face-to-face interventions. A number of previous studies showed that expressive writing assignments on specific bereavement-related themes can be effective in reducing the symptoms of PGD (81) because it enhances deep reflection on the loss, leads to new perspectives, or supports the process of sense-making (82). Only recently, Sloan and colleagues (83) compared a five session written exposure intervention with cognitive processing therapy (CPT), a first line treatment approach for PTSD. The results indicate that the written exposure intervention was equally effective as CPT in trauma-related symptom reductions. Expressive writing might enhance a deep reflection on the loss or the traumatic experience and might help to develop a narrative of it. The emotional processing during writing might finally lead to new perspectives that are perceived as less threatening. Further, writing might support the process of sense-making and relieve the distressed mourner (82). Taken together, the positive treatment effect of the analyzed internet-based interventions could be largely due to the written tasks. Hence, written assignments that foster self-disclosure and reduce avoidance seem worth considering in future online-interventions as well.

This review offers some practical implications. First, internet-based interventions are a promising alternative to treat PGD when face-to-face encounters are not possible or wanted. Because all reviewed interventions were based on CBT and mostly used writing assignments based on the Pennebaker paradigm (49), there seems to be evidence that clinicians can implement these structured writing approaches in their clinical practice. The main elements hereby are the examination of distressing remembrance using exposure elements, and cognitive restructuring of grief-related dysfunctional thoughts, as well as the integration and restoration (*i.e.*, development of new goals for the future). Although more research is still necessary to improve internet-based treatments for PGD, clinicians might pay special attention to very specific grief- and depression-related symptoms in addition to posttraumatic stress symptoms. Further, it seems advisable to offer online programs that are not too short (*i.e.*, >6 sessions) and to provide personal feedback and guidance to avoid frustration and early dropout.

In conclusion, the results suggest that internet-based treatments, based on CBT, can help to reduce the symptoms related to the loss of a significant person in a relatively short time. Yet, more qualitative RCTs with adequate power on that topic are needed.

## Data Availability Statement

The data that support the findings of this study are openly available in Open Science Framework at https://osf.io/7re5m/?view_only=66a8043dfe644b4699d619e03ee10c76.

## Author Contributions

BW: substantial contribution to the conception, drafting the work, and revising it critically for important intellectual content; final approval of the version to be published. UM: substantial contribution to every aspect of the manuscript, critical revision, interpretation of the data, and final approval and agrees to be accountable. LH: substantial contribution to interpretation of data, critical revision, and final approval and agrees to be accountable. NR: Initial substantial contribution to the conception and design of the work.

## Conflict of Interest

The authors declare that the research was conducted in the absence of any commercial or financial relationships that could be construed as a potential conflict of interest.

## Footnotes

1 Wagner et al. (18) report ESs for two IES subscales (*i.e.* Intrusion and Avoidance), which were combined into one ES for PTSD symptoms on the basis of a correlation of *r* = .59 (42, 53).
